# Designing Fuzzy Algorithms to Develop Healthy Dietary Pattern

**DOI:** 10.5812/ijem.9927

**Published:** 2013-07-01

**Authors:** Golaleh Asghari, Hanieh-Sadat Ejtahed, Mohammad Mahdi Sarsharzadeh, Pantea Nazeri, Parvin Mirmiran

**Affiliations:** 1 Nutrition and Endocrine Research Center, Obesity Research Center, Research Institute for Endocrine Sciences, Shahid Beheshti University of Medical Sciences, Tehran, IR Iran; 2 Department of Clinical Nutrition and Dietetics, Faculty of Nutrition and Food Technology, National Nutrition and Food Technology Institute, Shahid Beheshti University of Medical Sciences, Tehran, IR Iran

**Keywords:** Fuzzy Logic, Dietary Pattern, Food

## Abstract

**Background:**

Fuzzy logic, a mathematical approach, defines the percentage of desirability for recommended amount of food groups and describes the range of intakes, from deficiency to excess.

**Objectives:**

The purpose of this research was to find the best fuzzy dietary pattern that constraints energy and nutrients by the iterative algorithm.

**Materials and Methods:**

An index is derived that reflects how closely the diet of an individual meets all the nutrient requirements set by the dietary reference intake. Fuzzy pyramid pattern was applied for the energy levels from 1000 to 4000 Kcal which estimated the range of recommended servings for seven food groups including fruits, vegetables, grains, meats, milk, oils, fat and added sugar.

**Results:**

The optimum (lower attention – upper attention) recommended servings per day for fruits, vegetables, grain, meat, dairy, and oils of the 2000 kcal diet were 4.06 (3.75-4.25), 6.69 (6.25-7.00), 5.69 (5.75-6.25), 4.94 (4.5-5.2), 2.75(2.50-3.00), and 2.56 (2.5-2.75), respectively. The fuzzy pattern met most recommended nutrient intake levels except for potassium and vitamin E, which were estimated at 98% and 69% of the dietary reference intake, respectively.

**Conclusions:**

Using fuzzy logic provides an elegant mathematical solution for finding the optimum point of food groups in dietary pattern.

## 1. Background

A nutritious dietary pattern is an indispensable component of a healthy lifestyle, essential for promoting health and reducing the risk of major chronic diseases. Since all micronutrients are not distributed equally in foods, different kinds of foods need to be consumed daily. In recent years, several healthy patterns have been introduced such as the My Pyramid food guidance system (1), the Mediterranean dietary pattern (2), and the dietary approach to stop hypertension (DASH) (3); among these, the food guide pyramid was released as a simple tool to help people implement evidence-based dietary guidelines suitable for different levels of energy consumption (4). Beside the food guide pyramid developed by the United States Department of America (USDA), a few countries have introduced food guide pyramids for their own populations such as Japan or Canada (5, 6). The Japanese Food Guide Spinning Top is expressed in quantities of the “dish” rather than in the “food” format to estimate the quantity of food eaten in a daily diet (6); lower, in the Canadian food guide, the quantities along with the recommendations are suggested; the pyramid was developed based on modeling food groups and evaluation of simulating diets (5). Another method of diet optimization is using mathematical models i.e. linear programming to minimize deviations in food intake patterns between the observed and recommended diets (7). If all methods are properly designed, the food patterns can help individuals choose diets that have a high probability of meeting their nutrient needs, and a low risk of adverse effects from excessive intakes (8).

However, there is an undeniable problem in the My Pyramid guidance system; the recommended amount of food groups are absolute values and do not inform users about the deviation from healthy diet. The consequences of deviations from various food groups are different. For instance, if a person eats one unit extra from the oil group, it is more harmful than if he eats one unit extra from the vegetable group because vegetable is dense nutrient group and has low energy content. Therefore, it is hard to follow the My Pyramid exactly and subjects usually deviate from recommended values. To solve this limitation, we aim at introducing the fuzzy guideline that defines the percentage of desirability for the recommended amount of each food group. Fuzzy logic was initiated by Lotfi A. Zadeh et al. in 1965. The fuzzy logic method compared to conventional evaluations like true/false or yes /no system is a multi-value logic that an object can be a member of one set by the percentages of attendance deal with systems that cannot be defined precisely. Nutrient and food group requirements fall in to this category. Fuzzy sets are expanded to nutrition science (10-12). They represent optimal intake better than crisp sets for all nutrients, indicating that near the midpoint of the plateau area of the optimal intake range, health neither improves nor deteriorates as the intake of the nutrient is changed. Hence, there is an optimal intake range rather than a single optimal point of intake. The goal in nutrition education is to optimize the diet so that the requirements for all nutrients are met. Also, if nutritional intervention is necessary to optimize the diet, it is important that changes recommended in dietary habit not be too drastic to follow (12).

For a crisp set, elements of the set definitely do belong to the set; while in a fuzzy set, elements of the set have a degree of membership in the set. Crisp sets are subsets of fuzzy sets, which are sometimes not applicable for real expressions. For instance, an essential nutrient in a fuzzy set shows the degree of healthy dietary pattern when changing the intake of that nutrient and keeping the rest of diet constant at an optimal level; this model, which has no single optimal intake in which the healthy pattern is not changed, i.e. neither it is improved nor it is deteriorated, was broadly introduced by Wirsam in 1996 (12). Hence, to express uncertainty and imprecision, the fuzzy set is a necessary and powerful method. The membership function in fuzzy set is not absolute and is defined exactly, varying between 0 and 1 which represent the worst and the absolute optimum status, respectively. Therefore, to solve the limitation, the new fuzzy pyramid is introduced, facilitating a suitable membership function for each food group pattern intake.

## 2. Objectives

In the current study, we tried to design software which finds the best fuzzy dietary pattern that moderate energy and nutrient by the iterative algorithm.

## 3. Materials and Methods

### 3.1. Designing Fuzzy Dietary Pattern

In order to estimate the recommended servings of seven food groups including fruits, vegetables, grains, meats, milk, oils, and fat and added sugar, the average nutrient content of each food group and the thirty one energy and nutrient requirements of subjects should be determined. To calculate average nutrient content of each food group, the percentage of the total consumption of each food item in food groups should be multiplied by nutrient content of that food item. The weighted average and nutrient content of food groups was used from the 2005 My Pyramid (13). The recommended dietary allowance (RDA) was used to estimate nutrient requirements based on sex and age groups; indicating the recommended amount of a nutrient, i.e. in which 97-98% of people are in the healthy range (14-19). Moreover, the energy recommendation was calculated from the estimating energy requirement (EER) formula (20). These nutrients and energy should be met at the RDA and EER levels by consumption of seven food groups, respectively. Hence, to solve an optimization problem i.e. to satisfy RDA values, the fuzzy pyramid pattern was applied for the energy levels from 1000 to 4000 Kcal. For the current paper, 2000 Kcal energy dietary pattern was used as an example to introduce fuzzy pyramid pattern.

For calculating each nutrient intake of an individual, the consumption of each food group was multiplied by its average nutrient content; we then summed all amounts of previous calculations to achieve the total nutrient intake as shown in equation 1; ‘i’ stands for each nutrient and ‘f’, ‘v’, ‘g’, ‘m’, ‘d’, and ‘o’ stand for intakes of fruit, vegetable, grain, meat, dairy, and oil, respectively.

Following equations show steps of calculations:

**Equation (1):** Intake (i)= f × fruit nutrient (i) + v × vegetable nutrient (i) + g × grain nutrient (i) + m × meat nutrient (i) + d × dairy nutrient (i) + o × oil nutrient (i)

We defined the energy function as score in the following equation 2, computed for any values of consumption of each group.

$$\mathrm{Score}\left(f, v, g, m, d, o\right)=\overset{31}{\underset{i=1}{\sum }}F\left(i\right)$$

For all nutrients, whose standard is the RDA or the adequate intake (AI), F(i) is defined according to equation 3.

$$F\left(i\right)=\left\{\begin{array}{cc} 100 & intake_{\left(i\right)}\geq standard_{\left(i\right)}\\ \left(standard_{\left(i\right)}-intake_{\left(i\right)}\times 100\right) & intake_{\left(i\right)}<standard_{\left(i\right)} \end{array}\right\}$$

For carbohydrate, protein, fat, linoleic acid, and alpha linolenic acid, whose standards are the acceptable macronutrient distribution range (AMDR), F(i) is defined according to equation (4).

$$F\left(i\right)=\{\begin{array}{cc} 100 & {\text{lower standard range}}_{\left(i\right)}\leq {\text{intake}}_{\left(i\right)}\leq {\text{upper standard range}}_{\left(i\right)}\\ 100-\frac{\left({\text{lower standard range}}_{\left(i\right)}-{\text{intake}}_{\left(i\right)}\right)}{{\text{lower standard range}}_{\left(i\right)}}\times 100 & {\text{lower standard range}}_{\left(i\right)}>{\text{intake}}_{\left(i\right)}\\ 100-\frac{\left({\text{intake}}_{\left(i\right)}-{\text{upper standard range}}_{\left(i\right)}\right)}{{\text{upper standard range}}_{\left(i\right)}}\times 100 & {\text{intake}}_{\left(i\right)}>{\text{upper standard range}}_{\left(i\right)} \end{array}$$

For saturated fat and cholesterol, whose standards are the upper limit (UL) and 2005 dietary guidelines, F(i) is defined according to equation (5).

$$F\left(i\right)=\{\begin{array}{cc} 100 & {\text{Intake}}_{\left(i\right)}\leq {\text{standard}}_{\left(i\right)}\\ \left({\text{intake}}_{\left(i\right)}-{\text{standard}}_{\left(i\right)}\right)/\left({\text{standard}}_{\left(i\right)}\right)\times 100 & {\text{intake}}_{\left(i\right)}>{\text{standard}}_{\left(i\right)} \end{array}$$

For defining the fuzzy pattern, values of each food group intake were changed in the range of zero to double values of the MyPyramid guidance system per 0.5 serviExample of the 2000 Kcal Dietary Pattern Deviationng distance. The total score at the specific value of inExample of the 2000 Kcal Dietary Pattern Deviationtake was obtained by summation of all the possibilities of other groups’ intake. For instance, total score for intake of ‘i’ servings from vegetable group intake were computed according to equation (6):

$${\text{vgetable}}_{\text{totalscore}}\left(i\right)=\sum_{f=1}^{2\mathrm{Mypyramid(f)}}\sum_{g=1}^{2\mathrm{Mypyramid(g)}}\sum_{m=1}^{2\mathrm{Mypyramid(m)}}\sum_{d=1}^{2\mathrm{Mypyramid(d)}}\sum_{o=1}^{2\mathrm{Mypyramid(o)}}\text{score}\left(\mathrm{f, v, g, m, d, o}\right)$$

In equation (6), ‘v’ is equal to ‘i’.

After computation, total score of each food group was divided by the maximum total score of that group; in this way, the range of total score for each possibility was scaled between zero to one.

The values of score function, calculated based on F(i) summation, are between 0 to 3100. As the energy intake is more important than other nutrients, we decided on priori to weight energy more than others; however, to the best of our knowledge, the degree of importance for energy intake is not defined yet. Considering the weight of energy intake resulted in updating equation (2) to achieving equation (7).

$$\mathrm{Score}\left(f, v, g, m, d, o\right)=\overset{20}{\underset{i=1}{\sum F\left(i\right)+w\times F\left(\text{energy}\right)}}$$

**Figure 1.**
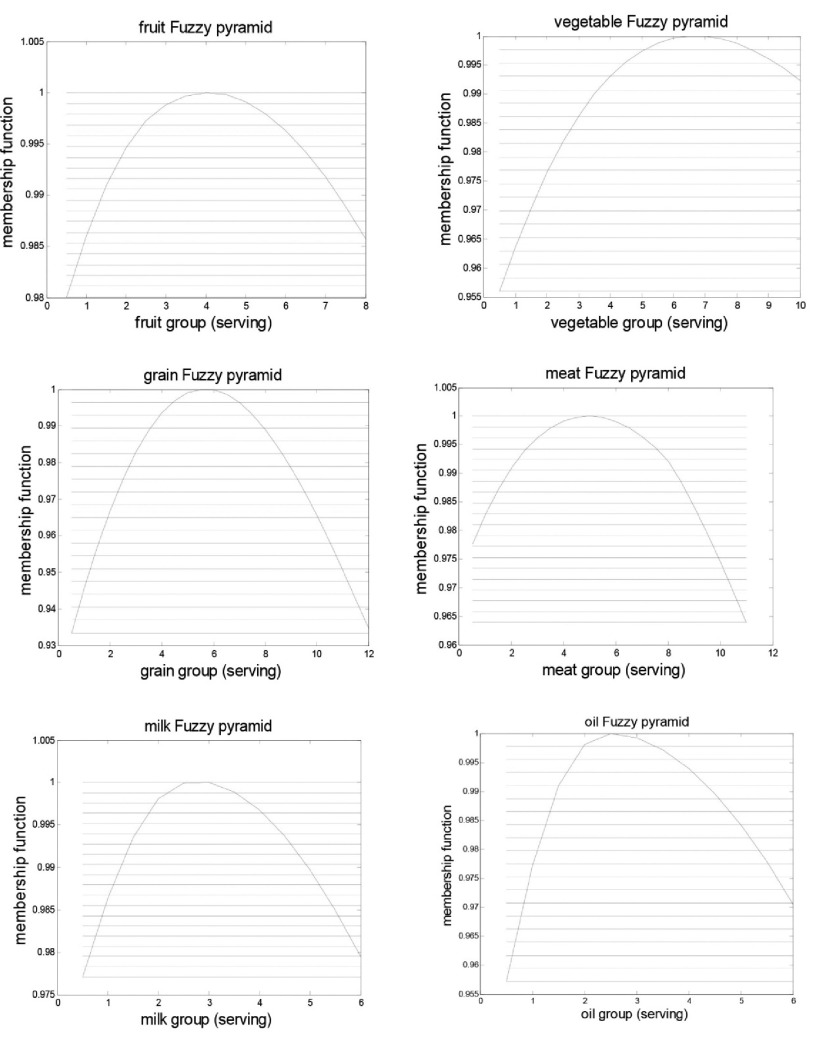
Fuzzy Set Curves for Six Food Groups of The 2000 Kcal Dietary Pattern

Therefore, for each value of six food group intakes between 0 to double values of MyPyramid with a 0.5 serving interval, the membership function was obtained and related curves were drawn (Figure 1). These curves provided the desirability for the amount of food groups’ intake which although applicable for researchers and professionals, are complicated for ordinary people.

**Figure 2.**
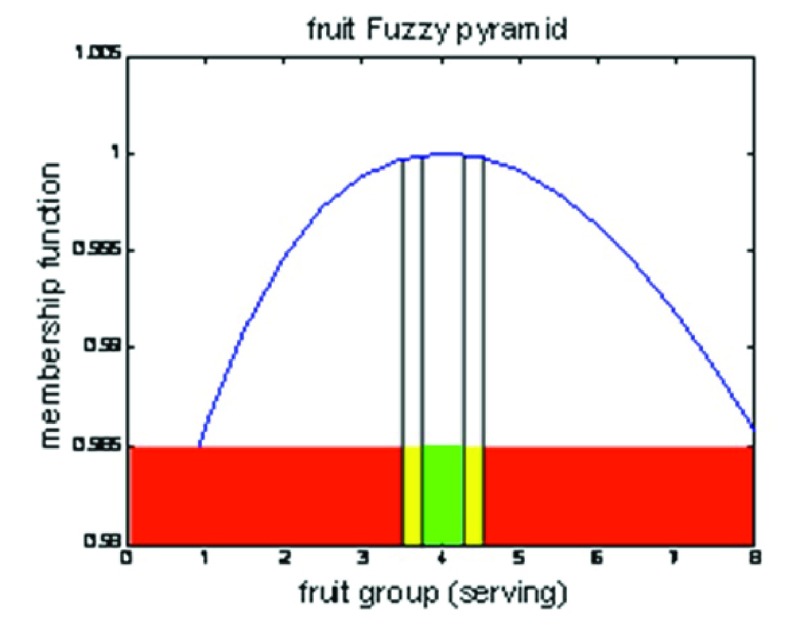
An Example of The 2000 Kcal Dietary Pattern Deviation

In order to simplify the current curves for ordinary people, we developed fuzzy man machine interface, which divides these curves into particular ranges: 1-Normal range (green color), 2- Attention range (yellow color), and 3- Danger range (red color) (Figure 2).

To find the upper and lower cut-points of these ranges, we designated 200 theoretical horizontal lines between the minimum to maximum scores of each food group curve, and the two intersecting points of these lines with the curve were obtained (Figure 1). Two lines were chosen according to the limitation of energy level to achieve three ranges. To find the “Normal range”, we considered a 5% deviation from the optimal energy level of the pattern as acceptable (1900-2100 calories). As mentioned above, since each line has two intersection points with curves, we had to choose a line, by which if the consumer eats the values of the first intersection point with intake curve, he receives 1900 calories and if he eats the values of the second point of intersection of that line with the intake curve, he receives 2100 calories. To find the “Attention range”, we considered the 5-10% deviation from the optimal energy level of the pattern as acceptable (1800-1900 and 2100-2200 calories); we had to choose a line, by which if the consumer eats the values of first intersection point with intake curve, he receives 1800 calories and if he eats the values of the second point of intersection of that line with intake curve, he receives 2200 calories; the space between the first point intersection of the second chosen line and the first point intersection of the first chosen line was considered as the “lower” Attention range, and the space between the second point intersection of the first chosen line and the second point intersection of the second chosen line was considered as the “upper” Attention range. The remaining area was considered as the “Danger range”. Afterward, we compared the obtained amounts of nutrients with their dietary reference intake (DRI) levels and these were reported as percentage of DRI (Table 1).

**Table 1.** Calculated Nutrient Content of 2000 kcal Dietary Fuzzy Pattern in Optimum Point and Different Borders of Intake

Nutrients	Danger and Attention Border		Attention and Normal Border		Optimum Point		Normal and Attention Border		Attention and Danger Border	
	amount	% DRI	amount	% DRI	amount	% DRI	amount	% DRI	amount	% DRI
**Energy,kcal**	1800		1900		2000		2100		2200	
**CHO, g**	242.7		255.8		270.0		284.2		299.5	
**CHO , % of calories**	51.5		51.5		51.5		51.5		51.5	
**Fiber , g**	26.8	95.6	28.0	100.1	29.6	105.7	31.2	111.3	33.0	118.0
**Protein , g**	76.9	167.1	82.3	179.0	88.5	192.3	94.6	205.6	100.7	218.8
**Protein ,% of calories**	17.2		17.0		17.5		18.0		18.0	
**Total fat , g**	62.2		66.3		68.9		71.5		75.1919	
**Total fat ,% of calories**	31.3		31.5		31		30.5		30.5	
**SFA , g**	17.8		18.6		19.2		19.8		20.6	
**SFA ,% of calories**	8.9		8.8		8.7		8.5		8.4	
**MUFA , g**	22.6		24.0		24.9		25.9		27.2	
**MUFA ,% of calories**	11.3		11.4		11.2		11.1		11.0	
**PUFA , g**	17.7		19.3		20.1		21.0		22.4	
**PUFA ,% of calories**	8.9		9.1		9.05		9.0		9.1	
**Linoleic acid , g**	15.9	99.4	17.3	108.1	18.1	113.1	18.8	117.5	20.1	125.6
**Alpha-** **linolenic** **, g**	1.6	88.9	1.8	100	1.85	102.8	1.9	105.5	2.0	111.1
**Cholesterol ,g**	189.0		200.8		214.3		227.8		239.6	
**Vitamin A ,** ** μg**	1086.9	133.1	1144.7	163.5	1213.5	173.4	1282.4	185.7	1363.9	194.8
**Vitamin E , μg**	9.1	60.8	9.8	65.3	10.3	68.6	10.8	72.0	11.5	76.9
**Vitamin C , mg**	150.7	183.2	159.6	212.8	169.7	226.3	179.9	239.8	191.3	255.1
**Thiamin , mg**	1.9	133.1	2.0	141.0	2.1	149.8	2.2	158.6	2.3	167.8
**Riboflavin , mg**	2.3	208.2	2.5	224.6	2.65	241.8	2.8	259.0	3.0	276.5
**Niacin ,mg**	19.4	138.6	20.5	146.4	21.7	155.3	23.0	164.3	24.2	173.2
**Vitamin B6 , mg**	2.0	152.0	2.1	160.1	2.2	169.7	2.3	179.3	2.5	189.7
**Folate** ** , μg**	681.5	170.4	716.0	179.0	756	189	796.0	199.0	840.9	210.2
**Vitamin B12** **, μg**	6.3	261.3	6.8	283.9	7.4	307.7	8.0	331.6	8.5	354.2
**Iron** **, mg**	16.6	92.3	17.4	96.9	18.4	102.2	19.4	107.6	20.4	113.4
**Calcium** **, mg**	1089.4	108.9	1185.0	118.5	1284.6	128.5	1384.3	138.4	1487.4	148.7
**Magnesium** **, mg**	350.8	113.2	372.2	120.1	396.6	127.9	421.0	135.8	447.4	144.3
**Zinc** **, mg**	12.1	150.7	12.9	160.7	13.7	171.9	14.7	183.3	15.6	194.5
**Phosphorus** **, mg**	1477.4	211.1	1588.3	226.9	1708.0	244.0	1827.8	261.1	1950.0	278.6
**Copper** **, mg **	1.4	160.9	1.5	169.7	1.6	180.1	1.7	190.5	1.8	201.9
**Potassium** **, mg**	3732.4	79.4	3982.2	84.7	4265.9	90.7	4549.6	96.8	4858.2	103.4

For added sugar and solid fats, we need to have moderation, which is in contrast to food groups for which we should have adequacy. Hence, the recommendation for the fuzzy pattern is different from other food groups; we had three ranges with three parts in contrast to food groups that had three ranges with five parts (Table 2), first, we chose the pick of the food group curves as the best point of recommendation and calculated the energy intake of this pattern for six groups; then, the difference between the energy intake and 2000 calories was considered as the Normal range of added sugar and solid fats, and 2000-2100 calories was considered as the Attention range. The remaining area was considered as the Danger range (Table 3).

**Table 2.** Normal, Attention and Danger Ranges of Six Food Group Intakes of the 2000 kcal Dietary Pattern

	Fruits	Vegetables	Grains	Milk	Meats	Oils
	**Lower**	**Lower**	**Lower**	**Lower**	**Lower**	**Lower**
**Danger and Attention Range**	3.5	6	5.5	2.25	4.2	2.2
**Attention and Normal Range**	3.75	6.25	5.75	2.5	4.5	2.5
**Normal and Attention Range**	4.25	7	6.25	3	5.2	2.75
**Attention and Danger Range**	4.5	7.5	6.5	3.25	5.5	3
	**Upper**	**Upper**	**Upper**	**Upper**	**Upper**	**Upper**

**Table 3.** Normal, Attention and Danger Ranges of Added Sugar and Solid Fats Group of the 2000 kcal Dietary Pattern

	Sugar and Fats
**Normal and Attention Bound**	270 Kcal
**Attention and Danger Bound**	370 Kcal
	Upper

## 4. Results

The fuzzy set curves for six food groups are shown in Figure 1. The optimum recommended serving per day for fruits, vegetables, grain, meat, dairy, and oil were 4.06, 6.69, 5.69, 4.94, 2.75, and 2.56, respectively.

Fuzzy sets have been developed to describe the range of intakes of a food group. The fuzzy set for fruits was almost symmetric, showing an optimum range recommending from 3.62 to 4.56 serving per day. As compared to a higher serving, scores of membership function decreased for lower serving, which is explained by the high nutrient density and fiber content of fruits. Vegetables and meat groups are the subject of considerable interest, which shows in two opposite direction. Only in the vegetable group, consumption over the recommendation point has the lowest reduction in the membership function and the gradient of the curve in lower serving intakes was more than higher serving intakes. However, overconsumption of meat is accompanied by the highest reduction in membership function, which indicates that the better desirability of consuming a lower serving, in comparison to a higher serving. One possible explanation might be the high content of saturated fatty acids and cholesterol in the meat group and the low calorie density, high fiber, water-soluble vitamins, phytochemicals, and mineral content of the vegetable group. The grain group shows steep slope on both sides with a narrow range, i.e. from 5.1 to 6.3, indicating that both lower and higher intakes, compared to the optimum, had similar effects. The oil and dairy groups had sharper curves than the others, indicating that wide deviations from optimum points are not allowed.

## 5. Discussion

Fuzzy logic theory is used as an approach in order to diminish errors establishing recommended intakes for nutrients or when evaluating dietary intake data (11). Similar to our results for a fuzzy pyramid dietary pattern, another study presented an optimal food plan using linear programming that complied with the recommended intake ranges for macronutrients, vitamins, and minerals and found the following results for food groups: Doubled amounts of fruit, reaching the upper range; increased milk, nuts, and proportions of all vegetable subgroups; exclusion of eggs, and lower amounts of meats, cheese and refined grains than the observed diets (7).

Table 1 shows the DRI for the entire nutrients of the 2000 fuzzy pattern, which met most recommended nutrient intake levels except for potassium and vitamin E, estimated at 98% and 69% of the DRI, respectively. These values were improved in comparison to the estimated intakes of the 1992 and 2005 food guide pyramid (21 ) and also the National Heart, Lung, and Blood Institute’s (NHLBI) Dietary Approaches to Stop Hypertension (DASH) Eating Plan, and the Harvard Healthy Eating Pyramid ( 22 ). This may be explained by the lack of rich sources of vitamin E such as nuts and potassium such as vegetables. In one of our previous studies, a higher DGAI score, suggesting better adherence to the dietary guidelines, demonstrated lower intake goals for potassium (4700 mg/dL) and vitamin E (15 mg/dL) ( 23 ). Greater specificity in food choices may be required to satisfy DRI values for vitamin E and potassium. Masset et al 2009 reported that to meet the requirements for all nutrients from the diet alone into a food plan on a daily basis, easier-to-find nutrients are provided in excess, findings similar to ours ( 7 ).

Previous studies have used mathematical optimization programming, such as linear and quadratic programming in nutrition (7, 21), which optimizes a linear or nonlinear function of decision variables, e.g. dietary patterns, while respecting the multiple constraints including cost and nutrient intake levels (24, 25). Gedrich et al compared the optimal diets computed with the non fuzzy approach with the optimal diets computed with the fuzzy approach. They concluded that non fuzzy approach satisfies all the persons' nutrient requirement; however make it necessary to change the persons' food habits considerably. Compared to the subjects' actual diets, the optimal diets computed with the fuzzy approach yield improvements for the intake of many nutrients, but in some cases such as folate, calcium and iodine deteriorations also occur (26).

In the current study, by changing ‘w’ as weight of energy, we observed that food groups’ curves would be changed. Higher values of ‘w’ were equaled to lower intake of high calorie food groups such as oils. Lower values of ‘w’ made the pick of curves to set on higher values of food groups, which would resulted in exceed of the energy intake levels. Using the iterative algorithm we tried to find the nearest pick of curves of food groups to the MyPyramid guidance system.

It is concluded that this software which introduces new fuzzy dietary pattern has some advantages. The desirability of each value of food group intake is defined in this pattern, and, it also provides a range of recommended servings for food groups, which makes this pattern a more applicable tool for population adherence. Finally, fuzzy dietary pattern supplies more nutrients needs in comparison to the MyPyramid pattern.

The limitation of this study is that this approach was not examined for confirmation of its validity in clinical settings. For the future, we intend to make medical evaluation studies to assert to which extent using this software and fuzzy dietary pattern can improve the users’ knowledge, and balances their dietary patterns.
